# 4-Benzyl-6-*p*-tolyl­pyridazin-3(2*H*)-one

**DOI:** 10.1107/S1600536809018376

**Published:** 2009-05-20

**Authors:** Ahmad Oubair, Jean-Claude Daran, Rachid Fihi, Lhou Majidi, Mohamed Azrour

**Affiliations:** a Laboratoire des Substances Naturelles et Synthèse et Dynamique Moléculaire, Faculté des Sciences et Techniques, BP 509, Errachidia, Morocco; b Laboratoire de Chimie de Coordination, UPR–CNRS 8241, 205 Route de Narbonne, 31077 Toulouse Cedex, France; c Laboratoire de Physico-Chimie des Matériaux, Faculté des Sciences et Techniques, BP 509, Errachidia, Morocco

## Abstract

The title compound, C_18_H_16_N_2_O, is a new dihydro­pyridazin-3(2*H*)-one derivative synthesized in one step by condensation of α-benzyl­idene-γ-tolyl­butenolide with hydrazine. The mol­ecule is not planar; the tolyl and pyridazine rings are twisted with respect to each other making a dihedral angle of 27.35 (9)° and the benzyl ring is nearly perpendicular to the pyridazine ring with a dihedral angle of 85.24 (5)°. In the crystal structure, inversion dimers arise, being linked by pairs of N—H⋯O hydrogen bonds. Weak C—H⋯O hydrogen bonds and weak offset π–π stacking stabilize the packing. The π–π stacking occurs between the pyridazine rings of symmetry-related mol­ecules, with a centroid–centroid distance of 3.748 Å, an inter­planar distance of 3.605 Å and a slippage of 1.024 Å.

## Related literature

For related compounds displaying biological activities, see: Sayed *et al.* (2002 ▶); Frolov *et al.* (2004 ▶); Piaz *et al.* (1994 ▶); Coelho *et al.* (2004 ▶); Malinka *et al.* (2004 ▶); Ogretir *et al.* (2002 ▶); Okcelik *et al.* (2003 ▶); Sotelo *et al.* (2003 ▶); Youssef *et al.* (2005 ▶). For related structures, see: Cao *et al.* (2003 ▶); Daran *et al.* (2006 ▶); Fihi *et al.* (1995 ▶); Filler & Piasek (1973 ▶); Roussel *et al.* (2000 ▶, 2003 ▶). For graph-set theory, see: Bernstein *et al.* (1995 ▶).
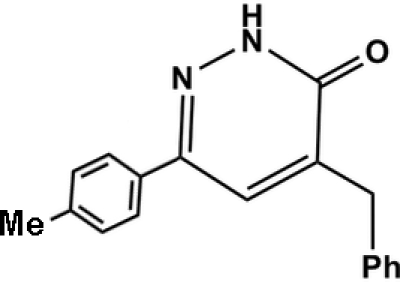


## Experimental

### 

#### Crystal data

C_18_H_16_N_2_O
*M* *_r_* = 276.33
Monoclinic, 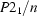

*a* = 7.2487 (4) Å
*b* = 10.4469 (5) Å
*c* = 19.1869 (9) Å
β = 99.598 (5)°
*V* = 1432.62 (12) Å^3^
*Z* = 4
Mo *K*α radiation
μ = 0.08 mm^−1^
*T* = 180 K
0.50 × 0.48 × 0.08 mm

#### Data collection

Oxford Diffraction Xcalibur diffractometer
Absorption correction: multi-scan (*CrysAlis RED*; Oxford Diffraction, 2006 ▶) *T*_min_ = 0.965, *T*_max_ = 0.993
10925 measured reflections
2914 independent reflections
1622 reflections with *I* > 2σ(*I*)
*R*_int_ = 0.044

#### Refinement

*R*[*F* ^2^ > 2σ(*F* ^2^)] = 0.041
*wR*(*F* ^2^) = 0.121
*S* = 0.94
2914 reflections
190 parameters
H-atom parameters constrained
Δρ_max_ = 0.20 e Å^−3^
Δρ_min_ = −0.20 e Å^−3^

### 

Data collection: *CrysAlis CCD* (Oxford Diffraction, 2006 ▶); cell refinement: *CrysAlis RED* (Oxford Diffraction, 2006 ▶); data reduction: *CrysAlis RED*; program(s) used to solve structure: *SIR97* (Altomare *et al.*, 1999 ▶); program(s) used to refine structure: *SHELXL97* (Sheldrick, 2008 ▶); molecular graphics: *ORTEPIII* (Burnett & Johnson, 1996 ▶), *ORTEP-3 for Windows* (Farrugia, 1997 ▶) and *PLATON* (Spek, 2009 ▶); software used to prepare material for publication: *SHELXL97*.

## Supplementary Material

Crystal structure: contains datablocks I, global. DOI: 10.1107/S1600536809018376/bg2256sup1.cif

Structure factors: contains datablocks I. DOI: 10.1107/S1600536809018376/bg2256Isup2.hkl

Additional supplementary materials:  crystallographic information; 3D view; checkCIF report

## Figures and Tables

**Table 1:** Hydrogen-bond geometry (Å, °)

*D*—H⋯*A*	*D*—H	H⋯*A*	*D*⋯*A*	*D*—H⋯*A*
N2—H2⋯O1^i^	0.88	1.89	2.7686 (19)	178
C34—H34⋯O1^ii^	0.95	2.57	3.512 (2)	169

Symmetry codes: (i) 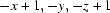; (ii) 
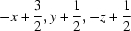
.

**Fig. 1.**
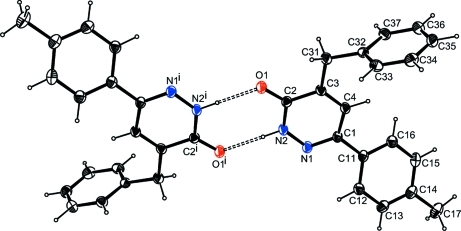
Molecular view of (I) with the atom-labelling scheme. Displacement ellipsoids are drawn at the 30% probability level. H atoms are represented as small spheres of arbitrary radii and hydrogen bonds are shown as dashed lines.[symmetry codes: (i) -x + 1, -y, -z + 1].

**Fig. 2.**
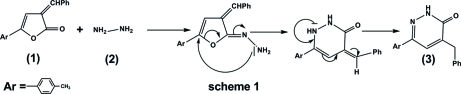
The formation of the title compound.

## supplementary crystallographic information

### Comment

In recent years a number of 6-arylpyridazin-3(2*H*)-ones have been reported to possess antimicrobial (Sayed *et al.*, 2002), anti-inflammatory (Frolov *et al.*, 2004), herbicidal (Piaz *et al.*,1994), antiplatelet activities (Coelho *et al.*, 2004), anticancer effects (Malinka *et al.*, 2004), antifeedant (Cao *et al.*, 2003), antihypertensive (Ogretir *et al.*,2002), potent analgesic (Okcelik *et al.*, 2003) and other anticipated biological(Youssef *et al.*, 2005) and pharmacological properties (Sotelo *et al.*, 2003).

In previous papers treating the reactivity of lactones bearing an exocyclic carbon-carbon double bond with 1,3-dipoles (Fihi *et al.*, 1995; Roussel *et al.*, 2000, 2003; Daran *et al.*, 2006), we reported that cycloaddition reactions lead to spiroheterocyclic compounds or evolutive products. In this paper, we describe the synthesis of a new dihydro-2 H– pyridazin-3-onederivative. The condensation ofα-benzylidene-γ-tolylbutenolide(1) and hydrazine (2) in reflux in toluene leads in one step to pyridazin-3-one(3). (Scheme).

Since the ^1^H and ^13^CNMR studies did not provide unambiguous information, a single-crystal of (3) was subjected to X-ray diffraction analysis to determine the structure of the product.

The molecule is not planar, the tolyl and the pyridazin rings are twisted to each other making a dihedral angle of 27.35 (9)° and the phenyl ring is nearly perpendicular to the pyridazin ring with a dihedral angle of 85.24 (5)° (Fig. 1).

The molecules are connected two by two through N—H···O hydrogen bonds with a *R*_2_^2^(8) graph set motif (Bernstein *et al.*, 1995) then building a pseudo dimer arranged around the inversion center (Fig. 1, Table 1). Weak C—H···O hydrogen bonds and weak offset π-π stacking stabilize the packing. The π-π stacking occurs between the pyridazin rings of symmetry related molecules with centroid-to-centroid distance of 3.748 Å and interplanar distance of 3.605Å and a slippage of 1.024 Å.

### Experimental

α-benzylidene-γ-tolylbutenolide (1) was synthesized according to the literature procedure (Filler & Piasek, 1973). (0.036 g, 1.125 mmol) of hydrazine was added to a solution of (1) (0.2 g, 0.76 mmol) in toluene (25 ml) and the mixture was stirred at reflux for 24 h. The solvent was then evaporated under reduced pressure. The residue was recrystallized from ethanol, and purified by chromatography on silica gel (eluant: ethyl acetate / hexane: 20 / 80). The pyridazinone was recrystallized from ethanol.

### Refinement

All H atoms attached to C atoms and N atom were fixed geometrically and treated as riding with C—H = 0.99 Å (methylene), 0.98Å (methyl) or 0.95 Å (aromatic) and N—H =0.88 Å with *U*_iso_(H) = 1.2*U*_eq_(C or N)or *U*_iso_(H) = 1.5*U*_eq_(C_methyl_).

### Crystal data

**Table:**

C_18_H_16_N_2_O	*F*(000) = 584
*M**_r_* = 276.33	*D*_x_ = 1.281 Mg m^−^^3^
Monoclinic, *P*2_1_/*n*	Mo *K*α radiation, λ = 0.71073 Å
Hall symbol: -P 2yn	Cell parameters from 3436 reflections
*a* = 7.2487 (4) Å	θ = 2.8–32.0°
*b* = 10.4469 (5) Å	µ = 0.08 mm^−^^1^
*c* = 19.1869 (9) Å	*T* = 180 K
β = 99.598 (5)°	Fragment, colourless
*V* = 1432.62 (12) Å^3^	0.50 × 0.48 × 0.08 mm
*Z* = 4	

### Data collection

**Table:**

Oxford Diffraction Xcalibur diffractometer	2914 independent reflections
Radiation source: fine-focus sealed tube	1622 reflections with *I* > 2σ(*I*)
graphite	*R*_int_ = 0.044
Detector resolution: 8.2632 pixels mm^-1^	θ_max_ = 26.4°, θ_min_ = 2.9°
ω and φ scans	*h* = −7→9
Absorption correction: multi-scan (*CrysAlis RED*; Oxford Diffraction, 2006)	*k* = −12→13
*T*_min_ = 0.965, *T*_max_ = 0.993	*l* = −23→23
10925 measured reflections	

### Refinement

**Table:**

Refinement on *F*^2^	Primary atom site location: structure-invariant direct methods
Least-squares matrix: full	Secondary atom site location: difference Fourier map
*R*[*F*^2^ > 2σ(*F*^2^)] = 0.041	Hydrogen site location: inferred from neighbouring sites
*wR*(*F*^2^) = 0.121	H-atom parameters constrained
*S* = 0.94	*w* = 1/[σ^2^(*F*_o_^2^) + (0.0651*P*)^2^ + 0.0125*P*] where *P* = (*F*_o_^2^ + 2*F*_c_^2^)/3
2914 reflections	(Δ/σ)_max_ = 0.008
190 parameters	Δρ_max_ = 0.20 e Å^−^^3^
0 restraints	Δρ_min_ = −0.19 e Å^−^^3^

### Special details

**Table:**

Experimental. All H atoms attached to C atoms and N atom were fixed geometrically and treated as riding with C—H = 0.93 Å (aromatic), 0.97 Å (methylene), 0.98Å (methyl) and N—H = 0.86 Å with *U*_iso_(H) = *xU*_eq_(C or N) where *x*=1.2 or 1.5(methyl).Empirical absorption correction using spherical harmonics, implemented in SCALE3 ABSPACK scaling algorithm, CrysAlis RED (Oxford Diffraction, 2006)
Geometry. All e.s.d.'s (except the e.s.d. in the dihedral angle between two l.s. planes) are estimated using the full covariance matrix. The cell e.s.d.'s are taken into account individually in the estimation of e.s.d.'s in distances, angles and torsion angles; correlations between e.s.d.'s in cell parameters are only used when they are defined by crystal symmetry. An approximate (isotropic) treatment of cell e.s.d.'s is used for estimating e.s.d.'s involving l.s. planes.
Refinement. Refinement of *F*^2^ against ALL reflections. The weighted *R*-factor *wR* and goodness of fit *S* are based on *F*^2^, conventional *R*-factors *R* are based on *F*, with *F* set to zero for negative *F*^2^. The threshold expression of *F*^2^ > σ(*F*^2^) is used only for calculating *R*-factors(gt) *etc*. and is not relevant to the choice of reflections for refinement. *R*-factors based on *F*^2^ are statistically about twice as large as those based on *F*, and *R*- factors based on ALL data will be even larger.

### Fractional atomic coordinates and isotropic or equivalent isotropic displacement parameters (Å^2^)

**Table:**

	*x*	*y*	*z*	*U*_iso_*/*U*_eq_	Occ. (<1)
O1	0.58992 (17)	−0.03563 (12)	0.42289 (6)	0.0387 (4)	
N1	0.8190 (2)	0.19591 (14)	0.54067 (8)	0.0336 (4)	
N2	0.7005 (2)	0.10629 (14)	0.50803 (7)	0.0335 (4)	
H2	0.6067	0.0848	0.5294	0.040*	
C1	0.9570 (3)	0.22886 (16)	0.50837 (9)	0.0296 (4)	
C2	0.7092 (3)	0.04582 (17)	0.44647 (9)	0.0297 (4)	
C3	0.8621 (2)	0.08373 (17)	0.41181 (9)	0.0296 (4)	
C4	0.9807 (2)	0.17325 (17)	0.44281 (9)	0.0309 (4)	
H4	1.0821	0.1999	0.4206	0.037*	
C11	1.0846 (3)	0.32907 (17)	0.54285 (9)	0.0314 (5)	
C12	1.0234 (3)	0.41994 (17)	0.58693 (9)	0.0360 (5)	
H12	0.8983	0.4168	0.5956	0.043*	
C13	1.1423 (3)	0.51460 (18)	0.61821 (10)	0.0398 (5)	
H13	1.0974	0.5757	0.6480	0.048*	
C14	1.3252 (3)	0.52198 (18)	0.60691 (10)	0.0392 (5)	
C15	1.3863 (3)	0.43020 (19)	0.56396 (10)	0.0414 (5)	
H15	1.5122	0.4326	0.5561	0.050*	
C16	1.2684 (3)	0.33473 (18)	0.53210 (9)	0.0372 (5)	
H16	1.3141	0.2731	0.5028	0.045*	
C17	1.4555 (3)	0.6246 (2)	0.64031 (11)	0.0547 (6)	
H17A	1.5780	0.6138	0.6259	0.082*	0.50
H17B	1.4691	0.6182	0.6919	0.082*	0.50
H17C	1.4044	0.7087	0.6250	0.082*	0.50
H17D	1.3897	0.6800	0.6693	0.082*	0.50
H17E	1.4986	0.6756	0.6033	0.082*	0.50
H17F	1.5633	0.5851	0.6702	0.082*	0.50
C31	0.8735 (3)	0.02039 (18)	0.34214 (9)	0.0359 (5)	
H31A	0.9169	−0.0688	0.3513	0.043*	
H31B	0.7463	0.0170	0.3138	0.043*	
C32	1.0013 (3)	0.08604 (17)	0.29952 (9)	0.0292 (4)	
C33	0.9342 (3)	0.18115 (18)	0.25246 (9)	0.0376 (5)	
H33	0.8063	0.2054	0.2473	0.045*	
C34	1.0500 (3)	0.2416 (2)	0.21276 (10)	0.0438 (5)	
H34	1.0024	0.3074	0.1806	0.053*	
C35	1.2341 (3)	0.20623 (19)	0.21994 (11)	0.0467 (6)	
H35	1.3142	0.2470	0.1923	0.056*	
C36	1.3030 (3)	0.11236 (19)	0.26684 (11)	0.0454 (6)	
H36	1.4309	0.0881	0.2719	0.054*	
C37	1.1874 (3)	0.05337 (18)	0.30651 (10)	0.0358 (5)	
H37	1.2363	−0.0111	0.3394	0.043*	

### Atomic displacement parameters (Å^2^)

**Table:**

	*U*^11^	*U*^22^	*U*^33^	*U*^12^	*U*^13^	*U*^23^
O1	0.0398 (8)	0.0418 (8)	0.0372 (8)	−0.0120 (7)	0.0141 (6)	−0.0062 (7)
N1	0.0369 (10)	0.0355 (9)	0.0299 (8)	−0.0082 (7)	0.0094 (7)	−0.0015 (7)
N2	0.0344 (9)	0.0379 (9)	0.0311 (8)	−0.0100 (8)	0.0139 (7)	−0.0040 (8)
C1	0.0326 (11)	0.0290 (10)	0.0281 (10)	−0.0032 (8)	0.0076 (8)	0.0052 (8)
C2	0.0331 (11)	0.0289 (11)	0.0278 (10)	−0.0029 (9)	0.0071 (8)	0.0012 (9)
C3	0.0306 (11)	0.0313 (11)	0.0285 (10)	0.0012 (8)	0.0093 (8)	0.0027 (8)
C4	0.0315 (11)	0.0336 (11)	0.0293 (10)	−0.0036 (9)	0.0100 (8)	0.0014 (9)
C11	0.0364 (12)	0.0327 (11)	0.0254 (9)	−0.0034 (9)	0.0064 (8)	0.0064 (9)
C12	0.0373 (12)	0.0382 (12)	0.0336 (11)	−0.0028 (9)	0.0088 (9)	0.0027 (10)
C13	0.0497 (14)	0.0344 (12)	0.0340 (11)	−0.0034 (10)	0.0034 (10)	0.0020 (9)
C14	0.0464 (13)	0.0354 (12)	0.0326 (11)	−0.0070 (10)	−0.0028 (9)	0.0077 (9)
C15	0.0336 (12)	0.0472 (13)	0.0420 (12)	−0.0084 (10)	0.0019 (9)	0.0116 (11)
C16	0.0395 (13)	0.0390 (12)	0.0341 (11)	−0.0023 (10)	0.0087 (9)	0.0041 (9)
C17	0.0584 (16)	0.0471 (14)	0.0534 (14)	−0.0177 (11)	−0.0058 (11)	0.0017 (11)
C31	0.0388 (12)	0.0403 (12)	0.0307 (10)	−0.0067 (9)	0.0119 (9)	−0.0046 (9)
C32	0.0354 (12)	0.0307 (11)	0.0227 (9)	−0.0041 (8)	0.0085 (8)	−0.0046 (8)
C33	0.0390 (12)	0.0408 (12)	0.0328 (11)	0.0043 (9)	0.0056 (9)	−0.0016 (9)
C34	0.0649 (16)	0.0374 (12)	0.0303 (11)	−0.0026 (11)	0.0111 (10)	0.0051 (9)
C35	0.0588 (16)	0.0452 (13)	0.0422 (12)	−0.0158 (11)	0.0257 (11)	−0.0073 (11)
C36	0.0353 (13)	0.0461 (13)	0.0575 (14)	−0.0009 (10)	0.0162 (11)	−0.0041 (12)
C37	0.0368 (12)	0.0366 (12)	0.0349 (11)	0.0014 (9)	0.0087 (9)	0.0002 (9)

### Geometric parameters (Å, °)

**Table:**

O1—C2	1.243 (2)	C16—H16	0.9500
N1—C1	1.308 (2)	C17—H17A	0.9800
N1—N2	1.3515 (19)	C17—H17B	0.9800
N2—C2	1.350 (2)	C17—H17C	0.9800
N2—H2	0.8800	C17—H17D	0.9800
C1—C4	1.422 (2)	C17—H17E	0.9800
C1—C11	1.478 (2)	C17—H17F	0.9800
C2—C3	1.440 (2)	C31—C32	1.500 (2)
C3—C4	1.341 (2)	C31—H31A	0.9900
C3—C31	1.506 (2)	C31—H31B	0.9900
C4—H4	0.9500	C32—C37	1.376 (3)
C11—C16	1.383 (3)	C32—C33	1.376 (2)
C11—C12	1.392 (2)	C33—C34	1.377 (3)
C12—C13	1.381 (2)	C33—H33	0.9500
C12—H12	0.9500	C34—C35	1.369 (3)
C13—C14	1.381 (3)	C34—H34	0.9500
C13—H13	0.9500	C35—C36	1.368 (3)
C14—C15	1.384 (3)	C35—H35	0.9500
C14—C17	1.500 (3)	C36—C37	1.369 (3)
C15—C16	1.387 (2)	C36—H36	0.9500
C15—H15	0.9500	C37—H37	0.9500
			
C1—N1—N2	116.10 (15)	C14—C17—H17D	109.5
C2—N2—N1	127.66 (15)	H17A—C17—H17D	141.1
C2—N2—H2	116.2	H17B—C17—H17D	56.3
N1—N2—H2	116.2	H17C—C17—H17D	56.3
N1—C1—C4	121.77 (17)	C14—C17—H17E	109.5
N1—C1—C11	116.46 (16)	H17A—C17—H17E	56.3
C4—C1—C11	121.76 (16)	H17B—C17—H17E	141.1
O1—C2—N2	120.52 (16)	H17C—C17—H17E	56.3
O1—C2—C3	124.21 (16)	H17D—C17—H17E	109.5
N2—C2—C3	115.27 (16)	C14—C17—H17F	109.5
C4—C3—C2	118.24 (16)	H17A—C17—H17F	56.3
C4—C3—C31	125.03 (17)	H17B—C17—H17F	56.3
C2—C3—C31	116.72 (16)	H17C—C17—H17F	141.1
C3—C4—C1	120.96 (17)	H17D—C17—H17F	109.5
C3—C4—H4	119.5	H17E—C17—H17F	109.5
C1—C4—H4	119.5	C32—C31—C3	114.42 (15)
C16—C11—C12	118.29 (17)	C32—C31—H31A	108.7
C16—C11—C1	120.67 (17)	C3—C31—H31A	108.7
C12—C11—C1	121.03 (17)	C32—C31—H31B	108.7
C13—C12—C11	120.87 (18)	C3—C31—H31B	108.7
C13—C12—H12	119.6	H31A—C31—H31B	107.6
C11—C12—H12	119.6	C37—C32—C33	118.48 (17)
C14—C13—C12	121.16 (19)	C37—C32—C31	121.19 (17)
C14—C13—H13	119.4	C33—C32—C31	120.33 (17)
C12—C13—H13	119.4	C32—C33—C34	120.83 (18)
C13—C14—C15	117.75 (18)	C32—C33—H33	119.6
C13—C14—C17	121.70 (19)	C34—C33—H33	119.6
C15—C14—C17	120.6 (2)	C35—C34—C33	119.65 (19)
C14—C15—C16	121.71 (19)	C35—C34—H34	120.2
C14—C15—H15	119.1	C33—C34—H34	120.2
C16—C15—H15	119.1	C36—C35—C34	120.14 (19)
C11—C16—C15	120.20 (18)	C36—C35—H35	119.9
C11—C16—H16	119.9	C34—C35—H35	119.9
C15—C16—H16	119.9	C35—C36—C37	119.89 (19)
C14—C17—H17A	109.5	C35—C36—H36	120.1
C14—C17—H17B	109.5	C37—C36—H36	120.1
H17A—C17—H17B	109.5	C36—C37—C32	121.01 (19)
C14—C17—H17C	109.5	C36—C37—H37	119.5
H17A—C17—H17C	109.5	C32—C37—H37	119.5
H17B—C17—H17C	109.5		
			
C1—N1—N2—C2	0.8 (3)	C12—C13—C14—C15	−0.9 (3)
N2—N1—C1—C4	−0.1 (2)	C12—C13—C14—C17	179.75 (17)
N2—N1—C1—C11	178.59 (14)	C13—C14—C15—C16	1.0 (3)
N1—N2—C2—O1	179.62 (16)	C17—C14—C15—C16	−179.62 (17)
N1—N2—C2—C3	−0.9 (3)	C12—C11—C16—C15	−0.9 (3)
O1—C2—C3—C4	179.72 (16)	C1—C11—C16—C15	179.14 (16)
N2—C2—C3—C4	0.3 (2)	C14—C15—C16—C11	−0.1 (3)
O1—C2—C3—C31	0.8 (3)	C4—C3—C31—C32	−14.1 (3)
N2—C2—C3—C31	−178.64 (15)	C2—C3—C31—C32	164.73 (16)
C2—C3—C4—C1	0.3 (3)	C3—C31—C32—C37	90.5 (2)
C31—C3—C4—C1	179.16 (17)	C3—C31—C32—C33	−89.3 (2)
N1—C1—C4—C3	−0.5 (3)	C37—C32—C33—C34	0.6 (3)
C11—C1—C4—C3	−179.06 (16)	C31—C32—C33—C34	−179.63 (17)
N1—C1—C11—C16	153.12 (17)	C32—C33—C34—C35	0.3 (3)
C4—C1—C11—C16	−28.2 (3)	C33—C34—C35—C36	−0.7 (3)
N1—C1—C11—C12	−26.8 (2)	C34—C35—C36—C37	0.2 (3)
C4—C1—C11—C12	151.86 (17)	C35—C36—C37—C32	0.7 (3)
C16—C11—C12—C13	1.0 (2)	C33—C32—C37—C36	−1.1 (3)
C1—C11—C12—C13	−179.02 (16)	C31—C32—C37—C36	179.14 (17)
C11—C12—C13—C14	−0.1 (3)		

### Hydrogen-bond geometry (Å, °)

**Table:**

*D*—H···*A*	*D*—H	H···*A*	*D*···*A*	*D*—H···*A*
N2—H2···O1^i^	0.88	1.89	2.7686 (19)	178
C34—H34···O1^ii^	0.95	2.57	3.512 (2)	169

Symmetry codes: (i) −*x*+1, −*y*, −*z*+1; (ii) −*x*+3/2, *y*+1/2, −*z*+1/2.
